# A Matron's Worries

**Published:** 1916-04-22

**Authors:** 


### THE EDITOR'S LETTER-BOX.

### A Matron's Worries.

*To the Editor of* The Hospital.

Sir,—I think "Matron of a Military Hospital" should try an O-Cedar mop for the ward floor. So long as the floor is cleaned with soap and water she will not be able to get a polished surface, and, of course, cleaning agents containing methylated spirit or turpentine are very expensive just now. The appearance of the floor would be greatly improved, at least for a time, if matron could raise the money to have the stained floor sized and varnished anew. The O-Cedar mop would preserve the polish and prevent the dusty appearance of which matron complains.—Yours truly, Another Matron.

